# Malondialdehyde as a Predictor of Disease Severity and Cardiovascular Risk in Population with Metabolic Dysfunction-Associated Steatotic Liver Disease

**DOI:** 10.3390/metabo16030203

**Published:** 2026-03-19

**Authors:** Roberto Lugo, Ana Ligia Gutiérrez-Solis, Ricardo Emmanuel Jimeno-Figueroa, Paul Góngora-Chan, Mayra Vera-Aviles, Dayana Williams-Jacquez, Marlene Chaurand-Lara, Jorge Arturo Valdivieso-Jimenez, Isabel Medina-Vera, Martha Guevara-Cruz, Brenda Pacheco-Hernández, Noriyouky Ix-Ruiz, Rodolfo Chim-Aké, Azalia Avila-Nava

**Affiliations:** 1 Unidad de Investigación, Hospital Regional de Alta Especialidad de la Península de Yucatán, Servicios de Salud del Instituto Mexicano del Seguro Social para el Bienestar (IMSS-BIENESTAR), Mérida 97130, Yucatán, Mexico; jlugo.hraepy@imssbienestar.gob.mx (R.L.); agutierrez.hraepy@imssbienestar.gob.mx (A.L.G.-S.); williamsdayana@gmail.com (D.W.-J.); dra.chaurand@gmail.com (M.C.-L.); brenda.pachecohernandez@gmail.com (B.P.-H.); kenori98.03@gmail.com (N.I.-R.); rodolfochim@hotmail.com (R.C.-A.); 2 Departamento de Radiología, Hospital Regional de Alta Especialidad de la Península de Yucatán, Servicios de Salud del Instituto Mexicano del Seguro Social para el Bienestar (IMSS-BIENESTAR), Mérida 97130, Yucatán, Mexico; jimeno.fig@gmail.com (R.E.J.-F.); medgeneral.drpaul@hotmail.com (P.G.-C.); 3 Department of Physiology, Anatomy and Genetics, University of Oxford, Sherrington Building, Parks Road, Oxford OX1 3PT, UK; mayra.veraaviles@dpag.ox.ac.uk; 4 Servicio de Medicina Interna, Hospital Regional de Alta Especialidad de la Península de Yucatán, Servicios de Salud del Instituto Mexicano del Seguro Social para el Bienestar (IMSS-BIENESTAR), Mérida 97130, Yucatán, Mexico; dr.arturo.valdivieso@gmail.com; 5 Departamento de Endocrinología, Hospital Regional Dr. Mario Cárdenas de la Vega, Instituto de Seguridad y Servicios Sociales de los Trabajadores del Estado (ISSSTE), Culiacán 80230, Sinaloa, Mexico; 6 Departamento Metodología de Investigación, Instituto Nacional de Pediatría, Ciudad de México 04530, Mexico; imedinav@pediatria.gob.mx; 7 Departamento de Fisiología de la Nutrición, Instituto Nacional de Ciencias Médicas y Nutrición Salvador Zubirán, Ciudad de México 14080, Mexico; martha.guevarac@incmnsz.mx

**Keywords:** malondialdehyde, oxidative stress, cardiovascular risks, steatosis

## Abstract

**Background/Objectives**: Metabolic dysfunction-associated steatotic liver disease (MASLD) is characterized by excessive triglyceride accumulation in the liver, the presence of one or more cardiometabolic risk factors, and an absence of harmful alcohol intake. Oxidative stress plays a crucial role in the development and severity of this disease, contributing to an increased cardiovascular risk (CVR). Malondialdehyde (MDA), an oxidative biomarker resulting from lipid peroxidation, is closely associated with metabolic dysfunction. This study aimed to evaluate the role of MDA as a predictor of steatosis severity and CVR. **Methodology**: An observational cross-sectional study was conducted in a population with MASLD with hepatic steatosis confirmed by ultrasonography and computed tomography. Subjects were classified according to severity of the hepatic steatosis as grade I or grade II-III. Nutritional, anthropometric, and serum biochemical parameters were measured. MDA levels were determined using a spectrophotometric method. The CVR was assessed using waist-to-hip ratio (WHR), triglycerides-glucose (TyG) index, lipid accumulation product (LAP), and atherogenic index of plasma (AIP). Receiver operating characteristic (ROC) curve analysis was performed to identify MDA cut-off value, followed by multivariable logistic regression to assess its association with severity of steatosis adjusted for body fat percentage. **Results**: A total of 50 patients were included (21 men and 29 women). An MDA cut-off value ≥ 0.13 nmol/mL was associated with higher severity (grade II–III vs. grade I) (OR = 5.0; 95% CI: 1.20–20.0; *p* = 0.022). Higher WHR values were found in subjects with grade I (*p* = 0.049), and elevated TyG index values were observed in patients with grade I-III (*p* = 0.042) both indicating increased CVR. **Conclusions**: Elevated MDA levels and higher body fat percentage were associated with higher degree of hepatic steatosis and increased CVR in the population from southeastern Mexico.

## 1. Introduction

Currently there are changes in eating habits, including an increase in refined carbohydrates and saturated fats. This excessive energy consumption has been linked to the development of pathologies such as metabolic dysfunction-associated steatotic liver disease (MASLD), formerly termed nonalcoholic fatty liver disease (NAFLD). It is the most common chronic liver disease worldwide [1].

The prevalence of MASLD worldwide is ~25–30% [2]. In Mexico, this prevalence is higher in comparation of other populations (~43%), and this may due to the high incidence of factors associated with its development, such as type 2 diabetes, and dyslipidemia [3,4]. In fact, this pathology is characterized by the excessive accumulation of fat in the liver, in the setting of metabolic dysfunction, including dyslipidemia and insulin resistance, which contribute to lipotoxicity, oxidative stress (OS) and chronic low-grade inflammation [5].

Altered lipid homeostasis and impaired mitochondrial β-oxidation promote metabolic inflexibility and increase the production of reactive oxygen species (ROS), leading to hepatic OS and lipotoxicity. This oxidative imbalance contributes to hepatocellular injury, inflammatory signaling, and activation of fibrogenic pathways, thereby facilitating disease progression from liver steatosis to advanced fibrosis, cirrhosis, and hepatocellular carcinoma [6].

The development of MASLD was previously explained by the “two-hit” hypothesis, which emphasized the roles of hepatic fat accumulation and OS in disease progression [7]. However, the currently accepted “multiple hits” hypothesis offers a more comprehensive understanding of MASLD pathophysiology. In this model, the “first hit” is driven by insulin resistance and metabolic dysfunction, leading to hepatic steatosis and liver injury through excessive fat accumulation and dysregulation of lipid metabolism, creating an imbalance between triglyceride uptake and elimination. Subsequent “hits” involve inflammation, apoptosis, fibrosis, OS, and hepatic lipid peroxidation. Lipid peroxidation occurs when ROS oxidize polyunsaturated fatty acids, generating a range of pro-inflammatory, fibrogenic, and oxidative metabolites that contribute to disease progression [8]. One of these metabolites is malondialdehyde (MDA), which serves as a commonly used biomarker of oxidative stress and can activate hepatic stellate cells, promoting collagen production and fibrosis [9,10].

Processes involved in MASLD, such as excess proinflammatory cytokines, OS, and increased biomarkers of oxidative damage, including MDA in plasma, have also been shown to predict cardiovascular risk (CVR) [11]. Emerging evidence suggests that MASLD itself constitutes an independent CVR factor in this population [12,13]. In fact, cardiovascular diseases are the leading cause of global mortality, and they represent approximately 80% of deaths occurring in low- and middle-income countries [14]. At national level, cardiovascular diseases are the leading cause of mortality and premature death in Mexico, representing over 25% of all deaths [15].

Therefore, identifying biomarkers such as MDA that enable early detection of oxidative damage in hepatic steatosis, during MASLD, is crucial for slowing disease progression and reducing associated complications, including CVR. Thus, the aim of this study was to evaluate MDA as a predictor of the steatosis severity and CVR.

## 2. Materials and Methods

### 2.1. Study Design and Population

A cross-sectional study with prospective recruitment was conducted at the Hospital Regional de Alta Especialidad de la Península de Yucatán, IMSS-BIENESTAR. The study included individuals suspected of having hepatic steatosis. Participants were excluded if they had a history of allergy to iodinated radiological contrast media, were undergoing chemotherapy and/or radiotherapy, were taking more than three medications associated with drug-induced liver injury or had a history of alcoholism or smoking. Additionally, individuals with human immunodeficiency virus infection, hepatitis B or hepatitis C virus infection, cirrhosis, or pregnancy were also excluded.

### 2.2. Sample Size

The sample size was determined based on a moderate-to-large effect size, aiming for a statistical power of 80% and a significance level of 0.05. A minimum of 46 participants was required.

### 2.3. Radiological Studies for Assess Hepatic Steatosis

All individuals underwent both computed tomography (CT) and ultrasonography (USG) to confirm the presence of hepatic steatosis. Hepatic steatosis is the hallmark of MASLD, characterized by the presence of hepatic steatosis alongside with at least one cardiometabolic risk factor and no other discernible cause [16]. Abdominal CT scans were performed using a 64-detector row scanner (General Electric Revolution EVO, GE HealthCare, Chicago, IL, USA). Acquisition parameters included a collimation of 5 mm, tube voltage of 120 kV, and tube current of 180 mA. Regions of interest (ROIs) were identified within homogeneous areas of the liver parenchyma and spleen. Each ROI covered approximately 20–30% of the evaluated area, and mean attenuation values were recorded in Hounsfield units (HU). Hepatic steatosis was defined as a mean hepatic attenuation value < 40 HU or a liver-spleen attenuation difference ≥ 10 HU. Images were viewed using RadiAnt DICOM Viewer software (version 4.6.4), with a soft-tissue abdominal window set to a window (window width: 400; window level: 50).

The USG studies were conducted using a Philips Affiniti 70 ultrasound system (Philips Healthcare, Andover, MA, USA) equipped with a convex transducer (5–10 MHz) operating in grayscale mode. Systematic sweeps were performed through the subxiphoid region in the sagittal plane to assess the left hepatic lobe, followed by evaluation of the right hepatic lobe via the right anterior axillary line, including measurement of its longitudinal diameter. Hepatic echogenicity was assessed by comparison with intrahepatic vascular structures and the ipsilateral diaphragm and, when possible, with the echogenicity of the splenic. Hepatic steatosis severity was graded according to a standardized USG classification system [17].

For both imaging techniques, examinations were interpreted by a specialized radiologist at a dedicated workstation using a medical-grade curved monitor with the Dicom Grayscale Standard Display Function (GSDF). Images were analyzed with a slide thickness of 2.5 mm and reconstructions of 1.25 mm.

### 2.4. General and Anthropometric Characteristics

Data on age, sex and blood pressure (BP) were collected from individuals diagnosed with hepatic steatosis. BP (systolic and diastolic) was measured after 15 min of rest in the sitting position using an automatic electronic sphygmomanometer (Omron, Hoffman Estates, IL, USA). Body weight (BW) and body composition, including fat mass, were determined using a multifrequency bioelectrical impedance analyzer (InBody 120, Seoul, Republic of Korea). Participants stood on the platform scale holding the device’s handles with both hands to provide contact with eight tetrapolar electrodes (two in each foot and two in each hand). Height was measured in centimeters using an electronic stadiometer (InLab S50, Seoul, Republic of Korea). Waist and hip circumferences were determined with a flexible measuring tape (Lufkin, Las Vegas, NV, USA) with an accuracy of 0.1 cm. All measurements were performed by trained personnel according to the method described by Lohman [18]. Body mass index (BMI) was calculated by dividing BW into Kg divided by the square of height in meters (Kg/m^2^).

### 2.5. Serum Biochemical Parameters

A trained researcher collected a blood sample after 10 h of overnight fasting. Samples were centrifuged at 1200× *g* for 10 min to separate serum and subsequently stored at −80 °C until analysis. Clinical biochemistry tests were measured using colorimetric assays with a Cobas c111 analyzer (ROCHE^®^ Cobas c111, Roche Diagnostics, Mannheim, Germany) to estimate levels of serum total cholesterol (TC) (mg/dL), triglycerides (TG) (mg/dL), high-density lipoprotein cholesterol (HDL-c) (mg/dL), low-density lipoprotein cholesterol (LDL-c) (mg/dL), and transaminase enzymes including alanine aminotransferase (ALT) and aspartate aminotransferase (AST).

### 2.6. Oxidative and Antioxidant Biomarkers

The serum concentration of MDA was measured using a spectrophotometric method [19]. Briefly, 200 μL of serum was mixed with 650 μL of 1-methyl-2-phenylindole (10 mM) and 150 μL of concentrated hydrochloric acid. The mixture was incubated at 45 °C for 40 min and centrifuged at 3000× *g* for 5 min. The optical density of the supernatant was measured at 586 nm, and a standard curve was constructed using tetramethoxypropane. MDA concentrations were expressed in nmol/mL.

Total antioxidant capacity (TAC) was assessed using the oxygen radical absorbance capacity (ORAC) assay [20]. Briefly, 25 μL of Trolox standards or serum (1:400) were mixed with 25 μL of 2,2-Azobis (2-methylpropionamidine) dihydrochloride (153 mM) and 150 μL of sodium fluorescein (50 nM) in 96-well black plates. Fluorescence was recorded at 390 nm excitation and 478 nm emission every minute for 90 min using a Synergy HT Multi-Mode Microplate Reader (BioTek Instruments, Inc., Winooski, VT, USA). TAC Results are expressed as Trolox equivalents (µmol/mL).

### 2.7. Cardiovascular Risk

We determined cardiovascular indices such as the waist-to-height ratio (WHR) [21] using the following formula:

$$WHR=\frac{WC\;(cm)}{Height\;(cm)}$$

the triglycerides-glucose (TyG) [22]:

$$TyG=Ln\left\lfloor \frac{Fasting\;TG\;(mg/dL)\times Fasting\;glucose\;(mg/dL)}{2}\right\rfloor$$

the triglyceride-to-high-density lipoprotein cholesterol ratio (TG/HDL-c) [23]:

$$TG/HDLc=\frac{TG\;(nmol/dL)}{HDLc\;(nmol/dL)}$$

the atherogenic index of plasma (AIP) [24]:

$$AIP={Log}_{10}\left\lfloor \frac{TG\;(nmol/dL)}{HDLc\;(nmol/dL)}\right\rfloor$$

and the lipid accumulation product (LAP) [25]:

$$LAP\;for\;women=[WC\left(cm\right)-65\times TG\;(nmol/L)]$$

$$LAP\;for\;men=[WC\left(cm\right)-58\times TG\;(nmol/L)]$$

The CVR was defined using the following cut-off values of each index: WHR > 0.5 [21], TG/HDL-c ratio ≥ 3.46 [26], TyG ≥ 4.49 [22], LAP ≥ 59.62, and AIP ≥ 0.107 [23].

### 2.8. Statistical Analysis

Normality of continuous variables was assessed using the Shapiro–Wilk test. Data are expressed as mean ± standard deviation or median and interquartile range [Q1, Q3], as appropriate. Comparisons between groups were performed using the unpaired Student’s *t*-test or Mann–Whitney *U*-test. Spearman’s rank correlation was used to assess the association between hepatic steatosis grades determined by USG and hepatic attenuation values measured in HU by CT. Receiver operating characteristic (ROC) curve analysis was performed to identify the optimal MDA cut-off value associated with disease severity. Additionally, a multivariable logistic regression model was constructed to assess the association between MDA levels and moderate-to-severe steatosis (grade II–III), adjusting for body fat percentage. Correlation between antioxidant levels (MDA) and CVR indices were performed using a Pearson’s *r* test. A *p*-value < 0.05 was considered statistically significant.

## 3. Results

**Figure 1:**
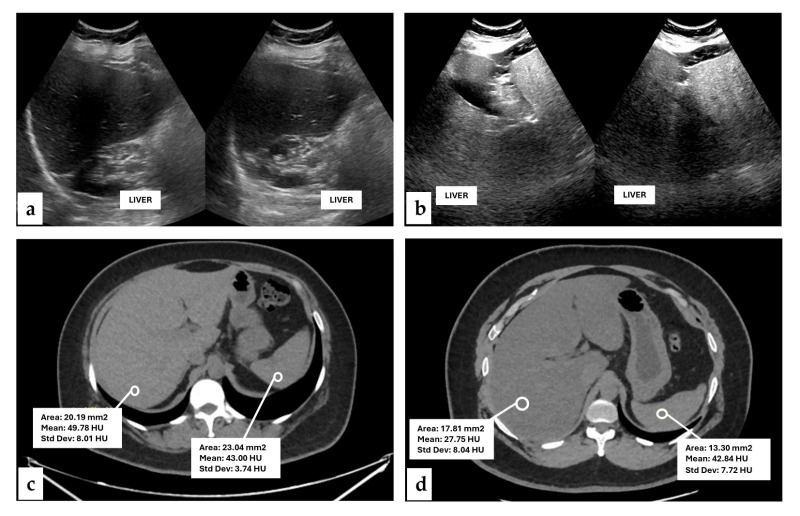
Representative USG images of hepatic steatosis: (**a**) USG Grade I and (**b**) USG Grade II–III. Corresponding CT images expressed in HU illustrated: (**c**) low-grade steatosis (49 HU) and (**d**) high-grade steatosis (27 HU).

A total of 50 individuals were included, with a mean age of 41.8 ± 11.8 years. Among the participants, 58% were women (*n* = 29) with a mean age of 42.0 ± 11.8 years. The subjects were classified according to the standardized USG grading system into grade I and grade II–III steatosis (Figure 1). The corresponding hepatic evaluation in HU for each USG grades was as follows: Grade I has a mean HU of 44.0 (39.17–47.00) while Grade II–III, had a HU mean of 29.5 (29.50–32.00). Spearman’s rank correlation analysis revealed a strong negative correlation between USG grades and HU attenuation values (Rho = −0.86, *p* < 0.001).

All individuals were residents of the Yucatan peninsula and were categorized into two groups according to their USG-determinate steatosis grade. Sociodemographic and body composition characteristics are detailed in Table 1. While most characteristics were similar between USG grade I and grade II–III, only body fat percentage and SBP differed significantly between the steatosis groups.

Sex-related differences in WC values were analyzed in individuals with low and high USG grades of hepatic steatosis. No statistically significant differences were observed in WC between USG Grade I and Grade II–III in males (111 ± 15.1 vs. 108 ± 16.1 cm; *p* = 0.775) or in females (94.1 ± 10.8 vs. 97.4 ± 6.60 cm; *p* = 0.344).

The biochemical parameters of the subjects did not differ significantly between groups (Table 2). Among these parameters, only aspartate aminotransferase (AST) was significantly higher grade I group (Table 2).

Levels of MDA and TAC were analyzed among USG steatosis grades. Table 3 shows a statistically significant difference in MDA levels, with increased values observed in USG grade II-III. In addition, no differences were observed in TAC.

ROC curve analysis was performed to determine the optimal MDA cut-off value associated with steatosis severity. An MDA value > 0.13 nmol/mg was identified, yielding a sensitivity of 58.3% and a specificity of 76.9% (Supplementary Figure S1). The median body fat percentage for the entire population was 45%, which was used as the cut-off point. Binary logistic regression analysis showed that body fat percentage ≥ 45% and MDA levels ≥ 0.13 nmol/mg were significantly associated with greater steatosis severity (grades II–III) (Table 4). The model demonstrated a good fit according to the Hosmer–Lemeshow test (χ^2^ = 0.653; *p* = 0.721) and explained 34.8% of the variance in liver steatosis (Nagelkerke R^2^ = 0.348), with an overall classification accuracy of 72%.

Since MASLD is defined as the presence of fat accumulation in the liver accompanied by excess of body weight, type 2 diabetes, dyslipidemias, and hypertension, among other factors, the presence of this disease may be confounded in women experiencing physiological changes associated with menopause. In this study, 58% of population were women; therefore, we analyzed MDA levels in premenopausal (*n* = 18) and postmenopausal (*n* = 11) subjects. In this context, we did not find statistically significant differences in MDA levels between premenopausal and postmenopausal women (0.356 [0.001–1.050] vs. 0.001 [0.001–1.87] nmol/mL; *p* = 0.981). However, when comparing TC (163 [126–180] vs. 204 [177–224] mg/dL; *p* = 0.031) and LDL-c (94.5 [70.0–123] vs. 144 [98.0–152] mg/dL; *p* = 0.026), statistically significant differences were observed between premenopausal and postmenopausal women, with higher values in those with USG grades II–III.

CVR in the population with MASLD was established using reference cut-off points for the following indices: WHR, TG/HDL-c, LDL-c/HDL-c, TyG index, LAP, and AIP. When comparing USG grades of hepatic steatosis, WHR showed higher values in USG grade I than in grades II–III. In contrast, the TyG index value was higher in USG Grade II–III (Table 5). Thus, both WHR and TyG index values exceeded the established reference cut-off points, suggesting an increased CVR.

Regarding the LAP index, there were no statistically significant differences between groups. However, when reference cut-off points for CVR were evaluated, subjects with USG grade II–III showed a 4.29-fold higher risk (95% CI: 1.14–16.1; *p* = 0.026) than those with USG grade I.

To determine the association between the MDA levels and cardiovascular indices, we analyzed the correlation between MDA and WHR, TyG, and LAP. No statistically significant differences were found between MDA and WHR (*r* = −0.160; *p* = 0.923), MDA and TyG (*r* = 0.588; *p* = 0.063), or MDA and LAP (*r* = 0.300; *p* = 0.838).

## 4. Discussion

Our results showed that elevated MDA levels and higher body fat percentage were associated with greater severity of hepatic steatosis and increased CVR, as assessed by the TyG and LAP indices, in the MASLD population. Metabolic comorbidities such as excess BW, dyslipidemia, and hypertension have been associated with MASLD, and influence its progression, including a higher likelihood of advanced fibrosis, cirrhosis, and hepatocellular carcinoma [27]. In our study, both steatosis groups exhibited excess BW as defined by BMI; however, BMI does not differentiate between lean and fat mass. Notably, body fat percentage was higher in the grade II–III group, consistent with previous studies showing that increased body fat is associated with a higher prevalence of MASLD [28,29]. This relationship is partly explained by the high lipolytic activity of adipose tissue, which releases free fatty acids directly into the portal circulation, leading to increased intrahepatic triglyceride accumulation [30]. Although circulating lipid profiles did not differ between groups, hepatic lipid accumulation assessed by ultrasound allowed stratification of steatosis severity.

During hepatic steatosis, dysregulation of oxidative metabolic processes promotes the formation of ROS resulting in systemic OS [31]. Under pathological conditions, redox imbalance damage biomolecules, including protein, DNA, and lipids. MDA is a biomarker of lipid oxidative damage in the liver and plays a key role in MASLD development and progression. Previous studies have shown that higher serum levels are associated with MASLD progression [32]. Moreover, the detection of MDA in liver biopsies from subjects with MASLD has been associated with a higher risk of having advanced fibrosis [33].

Oxidative damage is exacerbated by depletion of the endogenous antioxidant system. In fact, studies in subjects with MASLD have showed lower antioxidant and higher MDA levels [34], which are also associated with an increased risk of MASLD [OR: 1.51; 95% CI: (1.03–2.22); *p* = 0.034] [35]. These findings align with our results, in which elevated MDA levels [OR = 5.0; 95% CI: 1.20–20.00; *p* = 0.022] were associated with more severe steatosis. Regarding TAC, no significant differences were observed between groups. However, considering that antioxidant depletion may occur in early disease stages of MASLD, this reduction may contribute to increased oxidative damage in this type of pathology [36]. Although the increase in MDA levels, but not TAC levels, in advanced stages of hepatic steatosis may reflect that these biomarkers represent different components of the redox balance. MDA indicates ROS-mediated cellular damage, mainly associated with lipotoxicity, mitochondrial dysfunction, and increased hepatic β-oxidation, suggesting greater MASLD severity. In contrast, TAC might reflects the cumulative contribution of endogenous and dietary antioxidants and the scavenging capacity against peroxyl radicals; therefore, it may remain relatively stable despite increased oxidative stress or lipid damage [6,8,37].

As mentioned previously, MASLD is characterized by hepatic steatosis accompanied by non-communicable disease conditions and at least one cardiometabolic factor. However, these features may be confounded in women undergoing menopause. In our study, women were stratified according to the menopausal aged reported for the Mexican population (48 ± 4.3 years) [38], and MDA levels were analyzed. No statistically significant differences were observed between premenopausal and postmenopausal women. However, total cholesterol and LDL-c levels were higher in postmenopausal subjects. These findings are consistent with the study conducted by Grygiel-Górniak et al. [39], which reported an association between oxidative process, dyslipidemia, and nutritional behaviors in postmenopausal women, similar to the characteristics observed in our population, including poor dietary habits, excess of body weight and dyslipidemia.

Oxidative stress and hepatic fat accumulation have been linked to increased CVR through mechanisms such as endothelial dysfunction, a proinflammatory state, and accelerated atherogenesis. Various indices, including WHR, TG/HDL-C, LDL-C/HDL-C, TyG, LAP, and AIP, have been established to evaluate CVR. In our population, all indices indicated elevated CVR. Interestingly, WHR was higher in the grade I group, suggesting that early-stage steatosis may reflect adverse body fat distribution and dietary habits. The population from the Yucatan Peninsula is characterized by diets high in fats and simple carbohydrates, which contribute to hypertriglyceridemia, peripheral lipid accumulation, obesity, insulin resistance, and MASLD development [40,41]. Importantly, elevated WHR has been consistently reported in populations from the Yucatan Peninsula, indicating a high prevalence of central obesity independent of overall adiposity [42].

In contrast, the TyG and LAP indices were higher in the grade II–III group suggesting an increased risk of insulin resistance [21,25]. In the Mexican population, LAP values > 59.62 have been reported as a risk factor for insulin resistance [23]. MASLD frequently occurs in association with insulin resistance, with or without type 2 diabetes [43]. According to the National Health and Nutrition Survey (ENSANUT), type 2 diabetes affects 15.3% of adults over 20 years, while hypertension and obesity affect 12.9% and 35.3%, respectively, with slightly higher prevalence in southeast Mexico [3]. This region also presents a high burden of metabolic disorders, which may exacerbate hepatic steatosis severity and increase cardiovascular and cardiometabolic risk, as reflected by elevated MDA levels [44,45,46].

This study evaluated the associations between MDA levels and the WHR, TyG, and LAP indices. Although previous studies have suggested relationships between oxidative stress markers and CVR [19,41,47,48], no statistically significant correlations were observed in our analysis. Specifically, no association was found between WHR and MDA levels, which may be explained by the fact that lipid peroxidation is influenced by multiple metabolic factors beyond fat distribution. Similarly, the lack of correlation between LAP and MDA may reflect that oxidative damage is not determined solely by visceral fat accumulation but also by other metabolic disturbances, such as insulin resistance, dyslipidemia, and mitochondrial dysfunction.

Moreover, TyG and MDA levels showed a trend toward significance when the correlation between them was evaluated (*r* = 0.588; *p* = 0.063). In this context, high TyG levels may be associated with greater oxidative stress, more severe liver steatosis, metabolic inflammation, and increased levels of CVR. However, the lack of statistical significance in our study may be related to the limited sample size. Overall, these findings suggest that oxidative stress and CVR indices may reflect different but complementary aspects of metabolic dysfunction in MASLD.

Our findings highlight the role of MDA in hepatic steatosis severity and support the use of multiple indices for CVR assessment. However, this study has some limitations. Its cross-sectional design precludes causal inference, and hepatic steatosis was identified using imaging rather than biopsy; nevertheless, both CT and USG have high sensitivity. Monitoring MDA at different stages of hepatic steatosis in population with MASLD may provide additional insights. Finally, although the sample size was modest, stratification by steatosis grade allowed meaningful comparisons of biomarkers and CVR according to disease severity.

From a prospective standpoint, future research should focus on multicenter studies evaluating the clinical utility of MDA in patients at high CVR or with MASLD. Given the high global prevalence of type 2 diabetes and excess of body weight, MDA could serve as an early biomarker to identify individuals at greater risk of progression to conditions such as ischemic heart disease or chronic liver failure. In addition, integrating MDA measurements with imaging techniques such as ultrasound, computed tomography, or elastography, together with artificial intelligence approaches, may facilitate the development of clinical scores to identify patients at higher risk of disease progression. MDA could also be useful for monitoring responses to pharmacological and lifestyle interventions, allowing optimization of treatment strategies. If validated as an early biomarker, MDA may contribute to risk stratification, disease monitoring, and evaluation of treatment response in patients at increased risk of advanced liver disease, cardiovascular disease, and MASLD.

## 5. Conclusions

Elevated MDA levels and higher body fat percentage were associated with higher degree of hepatic steatosis and increased CVR, as assessed by WHR, TyG, and LAP indices, in the population with MASLD from southeastern Mexico. These findings reinforce the role of OS in disease progression and highlight the potential clinical utility of MDA as an accessible biomarker for identifying subjects at higher risk of advanced steatosis and cardiovascular complications.

## Figures and Tables

**Table 1:** General and anthropometric characteristics of the population with MASLD classified according to the USG grade of hepatic steatosis.

Characteristic	Grade I *n* = 26	Grade II–III *n* = 24	*p*-Value
Age (years)	43.1 ± 12.7	40.3 ± 10.7	0.339
Height (cm)	159 ± 11.5	155 ± 8.13	0.240
BW (kg)	86.3 ± 21.9	83.2 ± 13.4	0.555
BMI (kg/m^2^)	33.7 ± 5.54	34.3 ± 4.60	0.640
WC (cm)	103 ± 15.5	101 ± 11.1	0.553
HC (cm)	110 ± 11.0	112 ± 10.3	0.397
Body fat (%)	42.2 ± 5.99	46.0 ± 7.02	0.046
SBP (mm Hg)	126 ± 15.4	118 ± 17.9	0.034
DBP (mm Hg)	83.4 ± 8.70	82.0 ± 8.53	0.599

Data are presented as mean ± standard deviation. Statistical analysis was performed by unpaired Student’s *t*-test. All results were considered statistically significant at *p* < 0.05. BW: body weight; BMI: body mass index; WC: waist circumference; HC: hip circumference; SBP: systolic blood pressure; DBP: diastolic blood pressure.

**Table 2:** Serum biochemical parameters of the population with MASLD classified according to the USG grade of hepatic steatosis.

Characteristic	Grade I *n* = 26	Grade II–III *n* = 24	*p*-Value
Glucose (mg/dL)	90.3 [83.8–98.9]	94.1 [81.5–105]	0.404
Creatinine (mg/dL)	0.80 [0.69–1.04]	0.81 [0.67–0.95]	0.892
TG (mg/dL)	148 [106–175]	158 [107–211]	0.299
TC (mg/dL)	186 [159–202]	189 [163–224]	0.285
HDL-c (mg/dL)	47.7 [38.9–53.2]	48 [40.2–60.9]	0.491
LDL-c (mg/dL)	110 [79.4–142]	113 [84.0–149]	0.620
Serum total lipids (mg/dL)	259 [170–363]	240 [214–331]	0.969
ALT (U/L)	22.6 [17.4–42.9]	19.5 [17.2–26.3]	0.515
AST (U/L)	38.6 [23.5–46.8]	29.5 [17.7–35.5]	0.042

Data are presented as median [Q1, Q3]. Statistical analysis was performed by the Mann–Whitney *U*-test. All results were considered statistically significant at *p* < 0.05. TG: Triglycerides; TC: total cholesterol; HDL-c: high-density lipoprotein cholesterol; LDL-c: low-density lipoprotein cholesterol; ALT: alanine aminotransferase; AST: aspartate aminotransferase.

**Table 3:** Levels of oxidative stress biomarkers in population with MASLD classified according to USG grade of hepatic steatosis.

Characteristics	Grade I *n* = 26	Grade II–III *n* = 24	*p*-Value
MDA (nmol/mL)	0.01 [0.01–0.12]	0.54 [0.01–2.41]	0.021
TAC (µmol/mL)	2608 [1561–3156]	2195 [1632–2935]	0.442

Data are presented as median [Q1, Q3]. Statistical analysis was performed by the Mann–Whitney *U*-test. All results were considered statistically significant at *p* < 0.05. MDA: malondialdehyde; TAC: total antioxidant capacity.

**Table 4:** Association between MDA levels to predict grade of hepatic steatosis.

Variable	B	SE	OR [95% CI]	*p*-Value
Fat percentage > 45%	1.972	0.690	7.10 [1.86–27.7]	0.004
MDA > 0.13 nmol/mg	1.617	0.705	5.00 [1.20–20.0]	0.022

Statistical analysis was Binary logistic regression. All results were considered statistically significant at *p* < 0.05. B: coefficient of logistic regression; SE: standard error; OR: odds ratio; MDA: malondialdehyde.

**Table 5:** Cardiovascular indices in the population with MASLD stratified by USG grade of hepatic steatosis.

Characteristics	Grade I *n* = 24	Grade II–III *n* = 26	*p*-Value
WHR	0.935 ± 0.071	0.891 ± 0.06	0.049
TG/HDL-c	3.21 [2.90–4.02]	3.04 [2.40–4.40]	0.540
LDL-c/HDL-c	2.43 ± 0.85	2.43 ± 0.98	0.988
TyG	8.85 [8.47–8.96]	9.00 [8.62–9.17]	0.042
LAP	66.1 ± 25.0	75.9 ± 24.3	0.094
AIP	0.14 [0.02–0.23]	0.14 [0.04–0.27]	0.780

Data are presented as mean ± standard deviation or median [Q1, Q3]. Statistical analysis was performed by unpaired Student’s *t*-test or Mann–Whitney U-test. All results were considered statistically significant at *p* < 0.05. WHR: waist-to-hip ratio; TG/HDL-c: triglyceride-to-high-density lipoprotein cholesterol ratio; LDL-c/HDL-c: low-density lipoprotein cholesterol to high-density lipoprotein cholesterol ratio; TyG: triglyceride–glucose index; LAP: lipid accumulation product; AIP: atherogenic index of plasma.

## Data Availability

The original contributions presented in this study are included in the article and Supplementary Material. Further inquiries can be directed to the corresponding author. ed to the corresponding author.

## Supplementary Materials

The following supporting information can be downloaded at: https://www.mdpi.com/article/10.3390/metabo16030203/s1, Figure S1: ROC curve to determine the optimal MDA cut-off value associated with steatosis severity.

## Abbreviations

The following abbreviations are used in this manuscript:

MASLD	Metabolic dysfunction-associated steatotic liver disease
MDA	Malondialdehyde
WHR	Waist-to-hip ratio
TyG	Triglycerides-glucose
LAP	Lipid accumulation product
OS	Oxidative stress
ROS	Reactive oxygen species
CVR	Cardiovascular risk
CT	Computed tomography
USG	Ultrasonograpy
HU	Hounsfield units
BP	Blood pressure
BW	Body weight
WC	Waist circumference
HC	Hip circumference
BMI	Body mass index
TC	Total cholesterol
TG	Triglycerides
HDL-c	High-density lipoprotein cholesterol
LDL-c	Low-density lipoprotein cholesterol
TAC	Total antioxidant capacity
ROC	Area under the receiver operating characteristics curve
AIP	Atherogenic index of plasma
