# Supplementary figures and images for: Malondialdehyde as a Predictor of Disease Severity and Cardiovascular Risk in Population with Metabolic Dysfunction-Associated Steatotic Liver Disease
Source: Metabolites. 2026 Mar 19;16(3):203. doi: 10.3390/metabo16030203 (PMC13028847; doi:10.3390/metabo16030203)

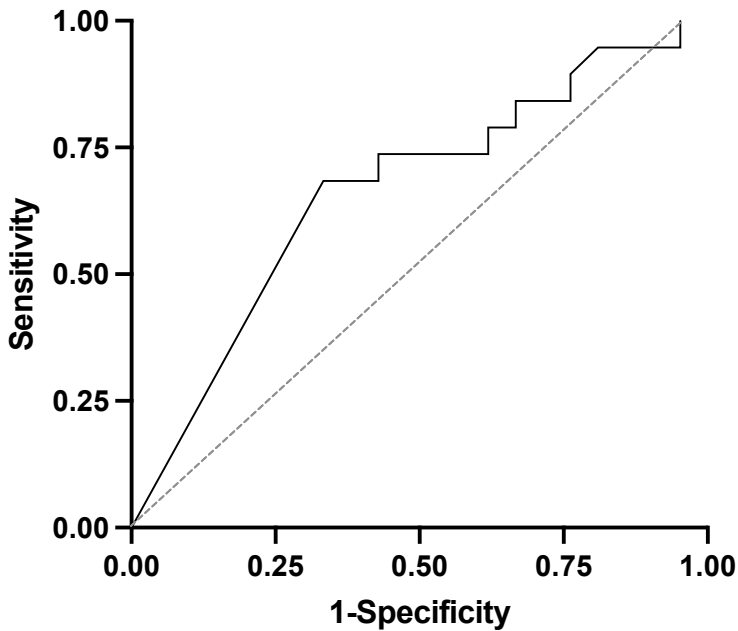

Supplement: Supplementary file 1 [file metabolites-16-00203-s001.zip › Figure S1.pdf]
